# OLSA-Pegelsteuerung bei monauraler Sprachaudiometrie im Störschall zur Evaluation des CI-Versorgungsergebnisses

**DOI:** 10.1007/s00106-022-01251-0

**Published:** 2022-12-05

**Authors:** Oliver C. Dziemba, Tobias Oberhoffner, Alexander Müller

**Affiliations:** 1 Klinik und Poliklinik für Hals‑, Nasen‑, Ohrenkrankheiten, Kopf- und Halschirurgie, Universitätsmedizin Greifswald, Ferdinand-Sauerbruch-Straße, 17489 Greifswald, Deutschland; 2 Klinik und Poliklinik für Hals-Nasen-Ohrenheilkunde, Kopf- und Halschirurgie „Otto Körner“, Universitätsmedizin Rostock, Rostock, Deutschland; 3 Klinik für Hals-Nasen-Ohrenheilkunde, Kopf- und Halschirurgie, Plastische Operationen, Vivantes Hörzentrum Berlin (HZB), Vivantes Klinikum im Friedrichshain, Berlin, Deutschland

**Keywords:** Sprachaudiometrie, Störschall, Pegelsteuerung, Hörversorgung, Sprachwahrnehmung, Speech audiometry, Noise, Level control, Hearing care, Speech perception

## Abstract

**Hintergrund:** Sprachaudiometrische Messungen unter Störschalleinflüssen sind grundlegender Bestandteil bei der Evaluation des Versorgungsergebnisses apparativer Hörversorgungen. Für die adaptive sprachaudiometrische Messung im Störschall bei Cochlea-Implantat(CI)-Trägern:innen existieren bisher noch keine Empfehlungen zur Wahl der Pegelsteuerungsmethode, d. h. entweder die adaptive Pegeländerung des Sprachsignals (S) bei konstantem Störgeräusch (N) oder die adaptive Pegeländerung von N bei konstantem S.

**Fragestellung:** Hat die verwendete Pegelsteuerung beim Oldenburger Satztest (OLSA) einen Einfluss auf die Ergebnisse der monaural gemessenen Sprachverständlichkeitsschwelle (SVS) im Störschall?

**Material und Methoden:** Insgesamt wurden von 50 CI-Trägern:innen die im Rahmen der klinischen Routine erzeugten OLSA-Messreihen im Störgeräusch mit unterschiedlicher Pegelsteuerung sowie sprachaudiometrischen Messungen in Ruhe mittels Freiburger Sprachtest ausgewertet und verglichen.

**Ergebnisse:** In Abhängigkeit von der Pegelsteuerung im OLSA zeigen sich keine signifikanten Unterschiede zwischen den ermittelten Sprachverständlichkeitsschwellen, die kleiner als 5 $\text{dB}_{\text{S/N}}$ sind. Unter 55 % Einsilberverständlichkeit im FBE wird die SVS im OLSA größer als 5 $\text{dB}_{\text{S/N}}$.

**Schlussfolgerungen:** Damit bei den Messungen mit positivem S/N der Summenpegel möglichst konstant gehalten wird bzw. nur wenig ansteigt, empfiehlt sich aus klinisch audiologischer und methodischer Sicht die Durchführung der adaptiven monauralen Sprachverständlichkeitsmessung mit konstantem Sprachsignal bei 65 $\text{dB}_{\text{SPL}}$. Zudem ist die Prüfung der monauralen Sprachverständlichkeit im Störschall erst ab einer Einsilberverständlichkeit von mindestens 55  % (65 $\text{dB}_{\text{SPL}}$) sinnvoll.

## Abstract (en)

**Scientific background:** Speech audiometry measurements under the influence of background noise are a fundamental part of evaluating the outcome of hearing care. As yet far, there are no recommendations for selecting a suitable method for adaptive speech audiometry measurements in background noise in cochlear implant (CI) care, so either the choice the adaptive level change of the speech signal (S) with constant noise (N) or the adaptive level change of N with constant S.

**Objectives:** Do the measurement results of the monaural speechrecognition threshold in noise (SRT) with the Oldenburg Sentence Test (OLSA) depend on the choice of level control?

**Material and methods:** A total of 50 series of measurements with OLSA in noise and the Freiburg speech intelligibility test in quiet (FBE) on middle-aged CI patients from clinical routine.

**Results:** There is no significant difference in the measurement results with different level controls when the SRT is less than 5 $\text{dB}_{\text{S/N}}$. Below 55 % monosyllabic intelligibility in quiet, the SRT in noise becomes greater than 5 $\text{dB}_{\text{S/N}}$.

**Conclusion:** From a clinical, audiological and methodological point of view, it is advisable to carry out the adaptive monaural speech intelligibility measurement with a constant speech signal at 65 $\text{dB}_{\text{SPL}}$.

## Einleitung

Sprachaudiometrische Messungen unter Störschalleinflüssen sind grundlegender Bestandteil bei der Evaluation des Versorgungsergebnisses apparativer Hörversorgungen [1, 2]. Nach den Empfehlungen der Deutschen Gesellschaft für Audiologie (DGA) [1] sollen sprachaudiometrische Untersuchungen im Störschall, der Belastungsfähigkeit des Patienten angepasst, im freien Schallfeld als einseitige Prüfung mit frontaler Darbietung von Sprache und Störschall ($\text{S}_{0^{\circ}}$/$\text{N}_{0^{\circ}}$) und als bilaterale Prüfung mit geeigneter Lautsprecheranordnung erfolgen. Nach dieser DGA-Empfehlung [1] können dafür verschiedene Sprachtestmaterialien verwendet werden. Eine wesentliche Anforderung an das Sprachmaterial besteht in dessen Evaluation, also dem Vorhandensein von Referenzwerten für den jeweiligen Einsatz im Störschall. In der klinischen Routine ist der Oldenburger Satztest (OLSA) bei Evaluationsmessungen von Hörsystemversorgungen im Störschall sehr weit verbreitet [3].

Der OLSA ist ein Matrixtest nach dem Vorbild von Hagermann 1982 [4], der für deutsche Sprache adaptiert, optimiert und für Messungen in Ruhe und im Störschall in einer Referenzsituation evaluiert wurde [5–7]. Neben statischen Messungen in Ruhe bei festen Darbietungspegeln oder im Störschall bei fixiertem Signal-Rausch-Abstand (S/N) können mit dem OLSA prozentuale Sprachverständlichkeitsschwellen (SVS) in Ruhe und im Störschall adaptiv gemessen werden. Die adaptive Messung einer 50 %-Sprachverständlichkeitsschwelle ($\text{L}_{50}$) im Störschall erfolgt dabei durch Variation des Darbietungspegels eines Signalanteils, wobei das jeweils andere Signal im Darbietungspegel fixiert bleibt [8]. Diese Pegelsteuerung stellt somit, neben der Wahl der Lautsprecheranordnung, einen zusätzlichen Freiheitsgrad dar. Aktuell existieren keine einheitlichen Empfehlungen zur Wahl der Pegelsteuerung bei Messungen im Störschall, sodass diese Parameterwahl in der Praxis sehr heterogen ist [3] und ein zentrumsübergreifender Vergleich der Evaluationsdaten erschwert ist. Analysen zur Qualitätssicherung bestimmter Hörsystemversorgungen aus speziell eingerichteten nationalen Registern, wie etwa einem Cochlea-Implantat(CI)-Register [2], können so unverhältnismäßig schwierig werden.

Wenngleich die Wahl der Pegelsteuerung bei adaptiven sprachaudiometrischen Messungen im Störschall auf den ersten Blick trivial erscheint, ergibt sich vor dem Hintergrund verschiedener Messmethoden und Fragestellungen die Notwendigkeit einer allgemeinen Empfehlung, um Messergebnisse verschiedener Institutionen möglichst vergleichbar zu halten. Für eine systematische Auswahl sprachaudiometrischer Verfahren existieren Empfehlungen, die das zu verwendende Sprachmaterial in Abhängigkeit von ökologischer Validität, Objektivität, Reliabilität und Sensitivität betrachten [9]. Derartige Empfehlungen zur Wahl der Pegelsteuerung bei adaptiven sprachaudiometrischen Verfahren für Messungen im Störschall sind in dieser Form eher rar. Bei der monauralen Sprachverständlichkeitsmessung im Störschall können Ansätze zur Bestimmung des maximal akzeptablen Störschalls (*engl. acceptable noise level*) [10] herangezogen werden.

Im Zentrum dieser Arbeit stand die Frage, ob die Messergebnisse der monauralen Sprachverständlichkeitsmessungen im Störschall mit dem OLSA von der Wahl der Pegelsteuerung abhängen. Es sollen Messergebnisse der Sprachverständlichkeitsschwelle im Störschall unter Verwendung unterschiedlicher Pegelsteuerung mit dem OLSA an CI-Träger:innen aus der klinischen Routine verglichen werden. Dafür wurde die Pegelsteuerung zur Messung nach erfolgter Trainingsphase geändert. Im Hinblick auf die zentrale Erfassung der CI-Versorgungsergebnisse in einem CI-Register [2] soll daraus einen Empfehlung für eine einheitliche Parameterwahl abgeleitet werden.

## Material und Methoden

Alle Messungen erfolgten im Rahmen der regulären Routinekontrolle. In diese Studie konnten 50 Messreihen an CI-Träger:innen verschiedener Versorgungsvarianten und unterschiedlicher Hörerfahrung eingeschlossen werden ($25\times$ links$/25\times$ rechts). Am Standort Greifswald konnten die Messungen von 28 CI-Träger:innen, an den Standorten Berlin und Rostock jeweils 11 CI-Träger:innen eingeschlossen werden. Das mittlere Alter der CI-Träger:innen betrug 53,7 Jahre (min. 15 Jahre; max. 81 Jahre). Die Hörerfahrung zur Messung lag im Mittel bei 37 Monaten. Einige CI-Träger:innen wurden im Rahmen der Routinekontrollen zu unterschiedlichen Zeitpunkten mehrfach gemessen. Eine Auswahl nach CI-Hersteller erfolgte nicht.

Die monauralen Sprachverständlichkeitsmessungn erfolgten mit dem Oldenburger Messprogramm OMA (HörTech gGmbH, Oldenburg, Deutschland) in der Version 1.5.5.0. Am Standort Greifswald wurde das Audiometer MA55 (MAICO Diagnostics GmbH, Berlin, Deutschland) und an den Standorten Rostock und Berlin das Audiometer AT900 oder AT1000 (AURITEC Medizindiagnostische Systeme GmbH, Hamburg, Deutschland) eingesetzt. Die Darbietung von Sprache und Störschall erfolgten im freien Schallfeld aus frontaler Richtung ($\text{S}_{0^{\circ}}$/$\text{N}_{0^{\circ}}$) aus einem Lautsprecher in 1 m Entfernung. Die Audiometrieräume erfüllten an allen Standorten nachweislich die Normforderung an maximal zulässigem Störschall und Nachhallzeit während der Messungen [11, 12].

Bei monauraler Sprachverständlichkeitsmessung mit CI in Ruhe als auch im Störschall wurde die jeweilige Gegenseite durch passive Vertäubung geblockt. Dabei wurden Ohrstöpsel Howard Leight Laser Lite® (Sperian Hearing Protection, San Diego, US-CA) und ein Peltor™ Optime II™ Kapselgehörschutz H520A (3M Deutschland GmbH, Neuss, Deutschland) verwendet.

Bei allen Messungen mit dem OLSA wurden Listen, bestehend aus 20 Sätzen verwendet. Eine OLSA-Messreihe bestand, wie in der klinischen Routine üblich, aus der Trainingsphase und der eigentlichen Messung. Alle Messreihen starteten mit fixiertem Messsignal bei 65 $\text{dB}_{\text{SPL}}$ und einem Signal-Rausch-Abstand von 15 $\text{dB}_{\text{S/N}}$. Zum Ausschluss von Trainingseffekten bei der Messung mit dem OLSA wurde die $\text{L}_{50}$-Messung so lange mit der gleichen Pegelsteuerung wiederholt, bis sich die Messergebnisse zweier, aufeinander folgender Messungen um weniger als die einfache Standardabweichung des SVS-Referenzwerts des OLSA im Störschall von 1,1 $\text{dB}_{\text{S/N}}$ unterschieden. Die letzte Messung konnte so als erste Studienmessung gewertet werden. Anschließend wurde eine $\text{L}_{50}$-Vergleichsmessung mit opponierter Pegelsteuerung durchgeführt.

Im Rahmen der CI-Routinekontrollen wurden ebenfalls sprachaudiometrische Messungen in Ruhe mit dem Freiburger Sprachtest durchgeführt. Der Hörverlust für Zahlwörter (HVZ), auch $\text{a}_{1}$-Wert genannt, und die prozentuale Sprachverständlichkeit des Freiburger Einsilbertests (FBE) bei Darbietungspegeln von 50 $\text{dB}_{\text{SPL}}$ (FBE@50dB), 65 $\text{dB}_{\text{SPL}}$ (FBE@65dB) und 80 $\text{dB}_{\text{SPL}}$ (FBE@80dB) wurden für eine vergleichende Analyse in die Auswertung (s. Diskussion) mit eingeschlossen. Zur Erhöhung der Genauigkeit des FBE wurden die Messungen pro Darbietungspegel mit zwei Testlisten nacheinander durchgeführt.

Die Zusammenstellung aller Messdaten erfolgte in MS Excel (Microsoft Corporation, Redmond, US-WA). Alle statistischen Analysen und grafischen Darstellungen wurden mit R und RStudio [13, 14] durchgeführt. Nach grafischer Analyse der Daten auf Normalverteilung durch die Darstellung im Histogramm erfolgte eine Signifikanzanalyse mittels gepaartem Student’s *t* Test. Das Signifikanzniveau wurde mit einem p‑Wert von $p<0{,}05$ festgelegt.

Die retrospektive Auswertung von Daten aus der klinischen Routine hörsystemversorgter Patienten wurde durch die Ethikkommission an der Universitätsmedizin Greifswald positiv beschieden (BB 049/17). Die Auswertung der Daten der beiden anderen Standorte erfolgte vollständig anonymisiert, sodass keine Zuordnung zu den Einzelpersonen mehr möglich war.

## Ergebnisse

**Figure 1:**
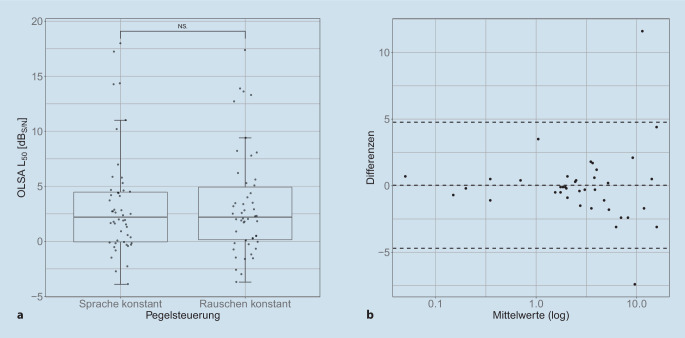

Die Lage und Streumaße der SVS im Störschall mit unterschiedlicher Pegelsteuerung sind in Abb. 1a dargestellt. Der Median der Messwerte bei konstantem Sprachpegel von 65 $\text{dB}_{\text{SPL}}$ liegt bei 2,2 $\text{dB}_{\text{S/N}}$ (oberes Quartil bei 4,5 $\text{dB}_{\text{S/N}}$). Der Median der Messwerte bei konstantem Störschallpegel von 65 $\text{dB}_{\text{SPL}}$ liegt ebenfalls bei 2,2 $\text{dB}_{\text{S/N}}$ (oberes Quartil bei 4,9 $\text{dB}_{\text{S/N}}$) und weist eine vernachlässigbar größere Streuung auf.

In Abb. 1b sind die Messergebnisse im Streudiagramm dargestellt. Zur Darstellung der Abhängigkeit der Streuung der Abweichung beider Pegelsteuerungsmethoden von der SVS wurde die Abszisse logarithmisch skaliert. In dieser Darstellung zeigt sich eine Schwankungsbreite der SVS im Störschall bei Messung mit unterschiedlicher Pegelsteuerung von $\pm$5 dB. Weiterhin stellt sich eine höhere Streuung der Messergebnisse mit steigender SVS dar.

**Figure 2:**
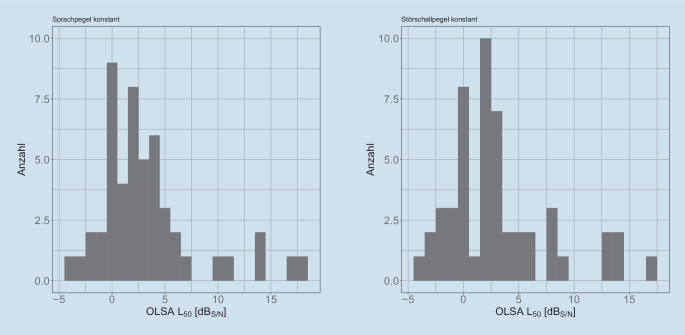

In Abb. 2 sind die Histogramme der Messergebnisse bei unterschiedlicher Pegelsteuerung dargestellt. Es zeigt sich eine Normalverteilung der $\text{L}_{50}$, insb. für Messwerte von $\text{L}_{50}<5$ $\text{dB}_{\text{S/N}}$. Unter der Annahme der Normalverteilung der Daten zeit der t.test keine signifikanten Unterschiede ($p=0{,}9164$) auf den 5 % Signifikanzniveau.

**Figure 3:**
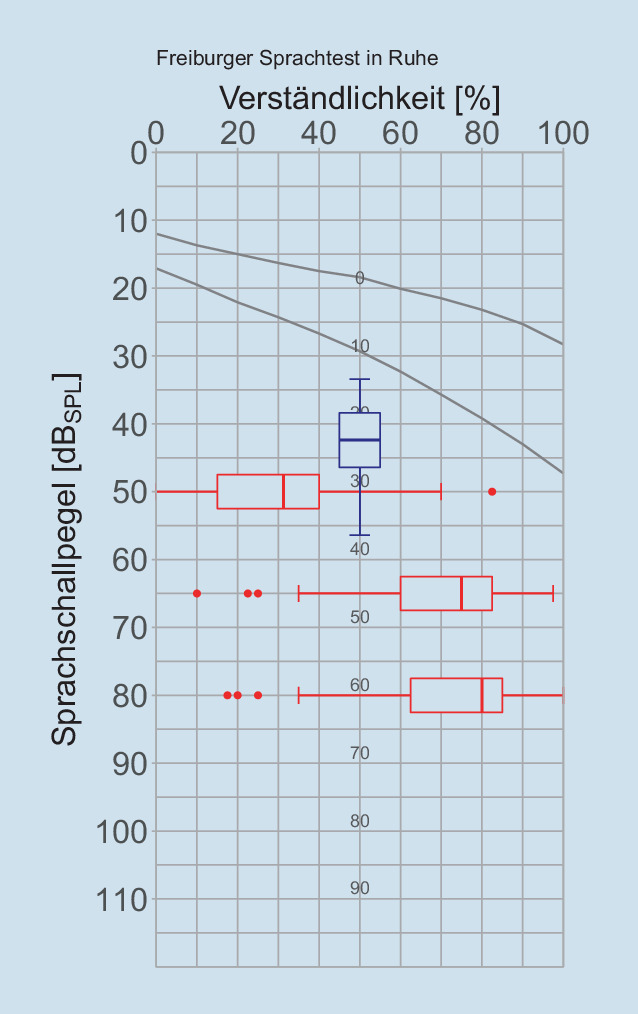

Die sprachaudiometrischen Ergebnisse in Ruhe, gemessen mit dem Freiburger Sprachtest, aller eingeschlossenen Messreihen sind in Abb. 3 dargestellt.

**Figure 4:**
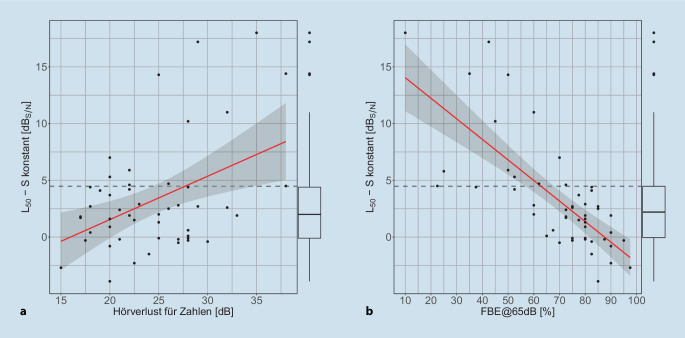

In Abb. 4 ist sie SVS, gemessen mit konstantem Sprachpegel von 65 $\text{dB}_{\text{SPL}}$, über dem HVZ und über der Einsilberverständlichkeit bei 65 $\text{dB}_{\text{SPL}}$ in Streudiagrammen dargestellt. Die lineare Regression zeigt in dieser Darstellung „SVS über HVZ“ auf Ebene des 75 %-Quartils aller SVS eine sehr hohe Streuung des Konfidenzintervalls von etwa 10 dB. In der Darstellung „SVS über FBE@65dB“ zeigt sich ein eher schmales Konfidenzintervall um die lineare Regression auf Ebene des 75 %-Quartils bei negativ korrelierten Werten.

## Diskussion

### Pegelsteuerung

Die zentrale Frage dieser Arbeit ist, ob die Pegelsteuerung beim Oldenburger Satztest (OLSA) einen Einfluss auf die Ergebnisse der monaural gemessenen Sprachverständlichkeitsschwelle (SVS) im Störschall hat.Dies wurde mittels OLSA bei CI-Träger:innen im Rahmen der Basis- und Folgetherapie analysiert. In der untersuchten Patientengruppe konnten keine signifikanten Unterschiede gefunden werden.

### Vergleichende Analyse

Bei der genauen Analyse der einzelnen Messergebnisse fällt auf, dass die Differenz beider Pegelsteuerungsmethoden ab einem Signal-Rausch-Abstand von etwa 5 $\text{dB}_{\text{S/N}}$ stark ansteigt. Dieses Ergebnis ist vergleichbar zu den Ergebnissen von Hey et al. [16]. Besonders bei CI-Träger:innen, deren Sprachverständlichkeit in Ruhe bei 65 $\text{dB}_{\text{SPL}}$ im OLSA unwesentlich besser als 50 % ist, ergeben sich unterschiedliche Ergebnisse in Abhängigkeit von der gewählten Pegelsteuerungsmethode. Ursächlich ist hier der entsprechende Sprach- und Störpegel und der daraus resultierende Summenpegel des gesamten Testsignals.

Besonders bei Sprachverständlichkeitsschwellen jenseits der 5 $\text{dB}_{\text{S/N}}$ zeigt sich ein erheblicher Unterschied im Summenpegel des Sprachtestmaterials. Bei der Testung mit konstantem Sprachsignal bei 65 $\text{dB}_{\text{SPL}}$ ergibt die Sprachverständlichkeitsschwelle von 5 $\text{dB}_{\text{S/N}}$ einen Summenpegel des Testmaterials von 66,2 $\text{dB}_{\text{SPL}}$ [17]. Bei einer Durchführung mit konstantem Rauschsignal bei 65 $\text{dB}_{\text{SPL}}$ würde bei gleicher Sprachverständlichkeitsschwelle ein Summenpegel des Sprachmaterials von 71,2 $\text{dB}_{\text{SPL}}$ resultieren [17]. Dies bedeutet, dass bei positivem S/N im $\text{L}_{50}$ bei einer Pegelsteuerung mit konstant gehaltenem Rauschpegel, der Pegel des Sprachsignals um die Größenordnung des S/N ansteigt und ebenso der Summenpegel des Sprachtests ansteigen würde. Somit würde das Testergebnis im OLSA im Störschall der individuellen Dikriminationsfunktion in Ruhe folgen, was eine interindividuelle Auswertung der Testergebnisse erschwert. Darüber hinaus würde eine Erhöhung des Gesamtpegels des Sprachtestsignals über 65 $\text{dB}_{\text{SPL}}$ die CI-Systeme des zu testenden Patienten bis weit in die Eingangspegelbegrenzung übersteuern und so zu weiteren Beeinträchtigungen des Messergebnisses beitragen.

Da bei der Durchführung des Oldenburger Satztests in der klinischen Routine Lern- bzw. Gewöhnungseffekte auszuschließen sind, sind die Messungen in der Regel zeitaufwendig und bedeuten für den untersuchten Patienten eine gewisse Anstrengung. Daher kann es wünschenswert sein, die Entscheidung zur Durchführung des OLSA im Störschall an die vorher durchgeführte Sprachaudiometrie in Ruhe zu knüpfen. Hier bietet sich der Freiburger Einsilbertest bei 65 $\text{dB}_{\text{SPL}}$ im freien Schallfeld mit CI an. Aus Abb. 4b kann abgeleitet werden, dass bei einer Einsilberverständlichkeit von weniger als 55 % nicht mehr mit einem $\text{L}_{50}$ im OLSA kleiner als 5 $\text{dB}_{\text{S/N}}$ gerechnet werden kann. Demzufolge ist aus Sicht der Autoren die Überprüfung der monauralen Sprachverständlichkeit im Störschall bei CI-Träger:innen erst ab einer Einsilberverständlichkeit in Ruhe von mindestens 55 % bei 65 $\text{dB}_{\text{SPL}}$ sinnvoll. Im Grenzfall wäre, beispielsweise während des Ausschlusses von Trainingseffekten im OLSA, eine Messung der Sprachverständlichkeit in Ruhe bei 65 $\text{dB}_{\text{SPL}}$ aufschlussreich. Sobald die individuelle Sprachverständlichkeit in Ruhe im OLSA bei 65 $\text{dB}_{\text{SPL}}$ unter 50 % sinkt, sind keine validen Ergebnisse adaptiver Messungen der Sprachverständlichkeit im Störschall bei konstantem Sprachpegel mit dem OLSA zu erwarten.

Vor dem Hintergrund von der Leistungsfähigkeit des individuellen Patienten [1] angepassten und zeitlich ökonomisierten audiometrischen Routinekontrollen von Hörsystemversorgungen, kann eine Staffelung der anzuwendenden Methoden erwogen werden. Mit dem Verweis auf noch nicht erreichte audiometrische Versorgungsziele weniger komplexer Messmethoden, wäre der Verzicht auf Methoden mit höherer Komplexität einfach zu begründen [9].

### Methodische Betrachtung

Die monaurale Sprachverständlichkeitsmessung im Störschall stellt insbesondere bei stark seitendifferentem Gehör oder einseitiger Taubheit eine gewisse Herausforderung dar. Diese Herausforderung besteht in der Wahl einer geeigneten Methode zum bestmöglichen Ausschluss von Überhören auf das nicht zu prüfende Gegenohr. Zur methodisch korrekten monauralen Messung im Störschall sollte aus Sicht der Autoren nur die Methode der passiven Vertäubung (Gehörstöpsel und Kapselgehörschutz) verwendet werden. Einschränkend besteht hier lediglich die Gefahr, dass die Wirkung der passiven Vertäubung durch einen variablen Gesamtpegel des Sprachtestmaterials im $\text{L}_{50}$ bei der Messung mit konstantem Rauschen nicht mehr ausreichend sein kann. Die Beurteilung des Messergebnisse im der klinischen Routine wäre in solchen Fällen erheblichen Unsicherheiten ausgesetzt.

Diese Betrachtungen beziehen sich ausschließlich auf die monaurale Messung der Sprachverständlichkeit im Störschall. Bei der binauralen Sprachverständlichkeitsmessung gelten in der Regel andere Anforderungen. Dies war aber nicht Gegenstand dieser Arbeit.

Aus Sicht der Autoren sollte somit die monaurale Messung der Sprachverständlichkeit mit dem Oldenburger Satztest mit konstantem Sprachpegel bei 65 $\text{dB}_{\text{SPL}}$ erfolgen. Diese Herangehensweise kann auch auf alternative Satztests, die für Messungen im Störschall evaluiert wurden, angewendet werden.

## Fazit für die Praxis

- Die Ergebnisse der adaptiven, monauralen Sprachverständlichkeitsmessung im Störschall mit dem Oldenburger Satztest (OLSA) zeigen keinen signifikanten Unterschied bei der Wahl der Pegelsteuerung und bei resultierenden Sprachverständlichkeitsschwellen $<5$ $\text{dB}_{\text{S/N}}$.
- Eine sichere und saubere Methode zur monauralen Messung der Sprachverständlichkeitsschwelle (SVS) im Störschall ist die Verwendung eines konstanten Sprachpegels von 65 $\text{dB}_{\text{SPL}}$, da sich an der Sprachverständlichkeitsschwelle bei positivem Signal-Rausch-Abstand ein vergleichbarerer Summenpegel des Testmaterials ergibt.
- Bei einer monauralen Einsilberverständlichkeit in Ruhe von weniger als 55 % bei 65 $\text{dB}_{\text{SPL}}$ kann auf die monaurale Messung im Störschall verzichtet werden.

## References

[1] Deutsche Gesellschaft für Audiologie e V. (2015). Audiologische Leistungen nach der CI-Indikation: Empfehlungen der Deutschen Gesellschaft für Audiologie (DGA). Z Audiol.

[2] Deutsche Gesellschaft für Hals-Nasen-Ohren-Heilkunde, Kopf- und Hals-Chirurgie e V (2021). Weißbuch Cochlea-Implantat(CI)-Versorgung: Empfehlungen zur Struktur, Organisation, Ausstattung, Qualifikation und Qualitätssicherung in der Versorgung von Patienten mit einem Cochlea-Implantat in der Bundesrepublik Deutschland.

[3] Baljić I, Wolf U, Sattler B (2020). Sprachaudiometrie in der CI-Nachsorge – Ergebnisse einer Umfrage. 51. Jahrestagung der DGMP : Abstractband.

[4] Hagerman B (1982). Sentences for testing speech intelligibility in noise. Scand Audiol.

[5] Wagener KC, Kühnel V, Kollmeier B (1999). Entwicklung und Evaluation eines Satztests für die deutsche Sprache I: Design des Oldenburger Satztests. Z Audiol.

[6] Wagener KC, Brand T, Kollmeier B (1999). Entwicklung und Evaluation eines Satztests für die deutsche Sprache Teil II: Optimierung des Oldenburger Satztests. Z Audiol.

[7] Wagener KC, Brand T, Kollmeier B (1999). Entwicklung und Evaluation eines Satztests in deutscher Sprache III: Evaluation des Oldenburger Satztests. Z Audiol.

[8] Brand T, Kollmeier B (2002). Efficient adaptive procedures for threshold and concurrent slope estimates for psychophysics and speech intelligibility tests. J Acoust Soc Am.

[9] Steffens T (2017). Die systematische Auswahl von sprachaudiometrischen Verfahren. HNO.

[10] Dillon H (2012). Hearing aids.

[11] Deutsches Institut für Normung e V. DIN EN ISO 8253-3:2012-08, Akustik – Audiometrische Prüfverfahren – Teil 3: Sprachaudiometrie (ISO 8253-3:2012); Deutsche Fassung EN ISO 8253-3:2012. Berlin: Beuth Verlag GmbH;. 10.31030/1861048

[12] Müller A, Mir-Salim P, Dziemba OC, Kirchner T (2018). Messung und Beurteilung von Ruhe- und Störschallpegeln in Hörprüfkabinen. Z Audiol.

[13] R Core Team (2020). R: a language and environment for statistical computing.

[14] RStudio Team (2021). Rstudio: integrated development environment for R.

[15] Deutsches Institut für Normung e V. (1995). Tonträger mit Sprache für Gehörprüfung Teil 1: Tonträger mit Wörtern nach DIN 45621‑1 (Aufnahme 1969).

[16] Hey M, Hocke T, Hedderich J, Müller-Deile J (2014). Investigation of a matrix sentence test in noise: Reproducibility and discrimination function in cochlear implant patients. Int J Audiol.

[17] Mrowinski D, Scholz G, Steffens T (2017). Audiometrie: Eine Anleitung für die praktische Hörprüfung.

